# Supplementary material for: Raphani Semen (Raphanus sativus L.) Ameliorates Alcoholic Fatty Liver Disease by Regulating De Novo Lipogenesis
Source: Nutrients. 2021 Dec 13;13(12):4448. doi: 10.3390/nu13124448 (PMC8705906; doi:10.3390/nu13124448)
Supplement: Supplementary file 1 [file nutrients-13-04448-s001.zip › nutrients-1441813-supplementary.pdf]

Supplementary Information

Raphani Semen (Raphanus sativus L.) ameliorates alcoholic fatty liver disease by regulating de novo lipogenesis

Park et al.

Figure S1. Effect of RSWE on p-IκBα and NF-κB in liver of C57BL/6J NIAAA mouse model.

Figure S2. Effect of RSWE on ATGL, HSL, and CPT1β in liver of C57BL/6J NIAAA mouse model.

Figure S3. Effect of RSWE on ACC in liver of C57BL/6J NIAAA mouse model.

Figure S1.

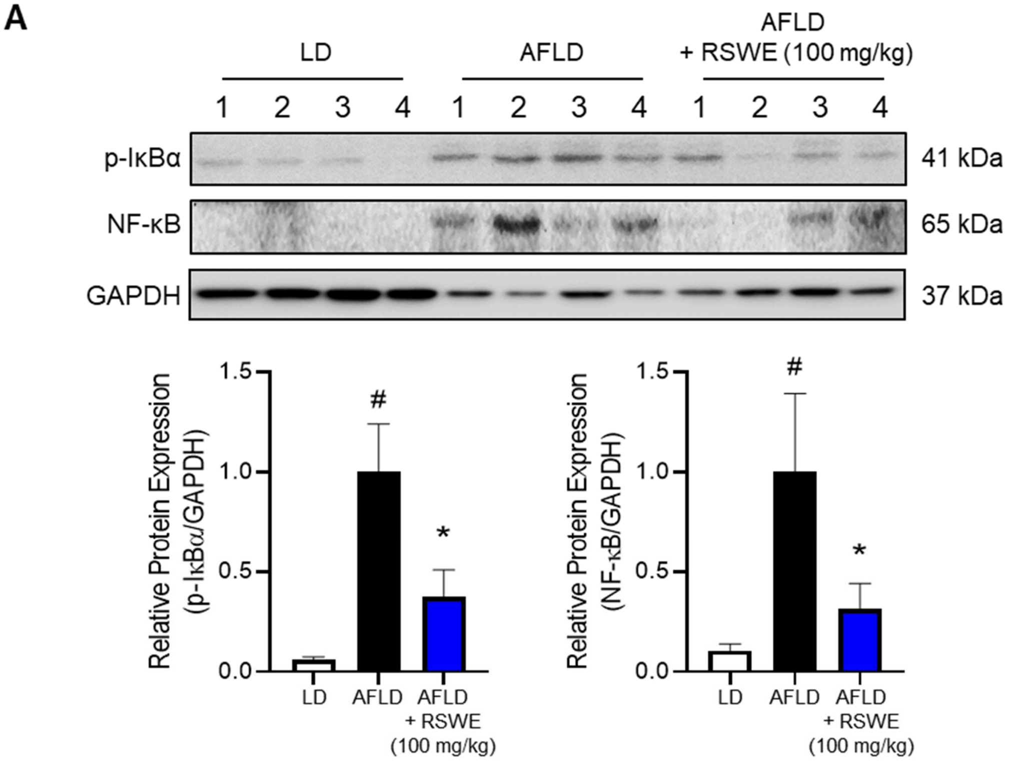

Figure S1. Effect of RSWE on p-IκBα and NF-κB in liver of C57BL/6J NIAAA mouse model.

(A) Protein levels of p-IκB, and NF-κB were analyzed using Western blot analysis. GAPDH was used as a loading control. All data are expressed as the mean ± S.E.M. of data (*n* = 4, per group). $^{\#}p < 0.05$ vs. LD group, $^{*}p < 0.05$ vs. AFLD group. RSWE, Raphani Semen water extract; LD, liquid diet; AFLD, alcoholic fatty liver diseases.

Figure S2.

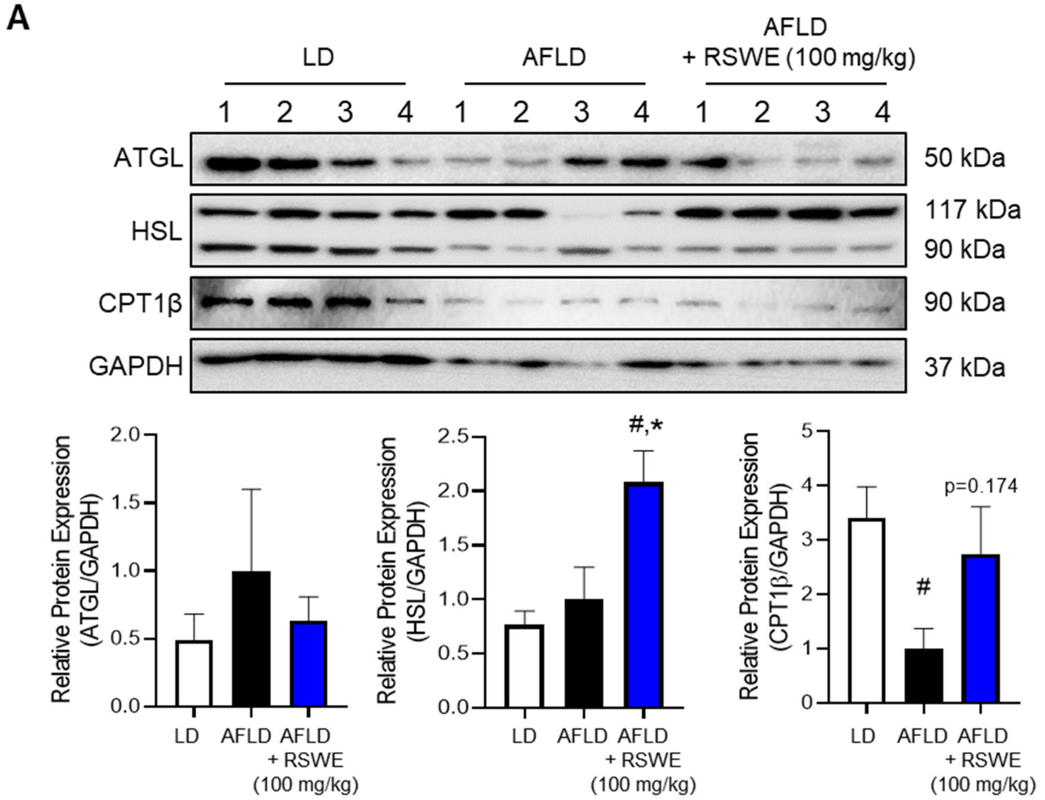

Figure S2. Effect of RSWE on ATGL, HSL, and CPT1β in liver of C57BL/6J NIAAA mouse model.

(A) Protein levels of ATGL, HSL, and CPT1β were analyzed using Western blot analysis. GAPDH was used as a loading control. All data are expressed as the mean ± S.E.M. of data (n = 4, per group). $^{\#}p < 0.05$ vs. LD group, $^{*}p < 0.05$ vs. AFLD group. RSWE. RSWE, Raphani Semen water extract; LD, liquid diet; AFLD, alcoholic fatty liver diseases.

Figure S3.

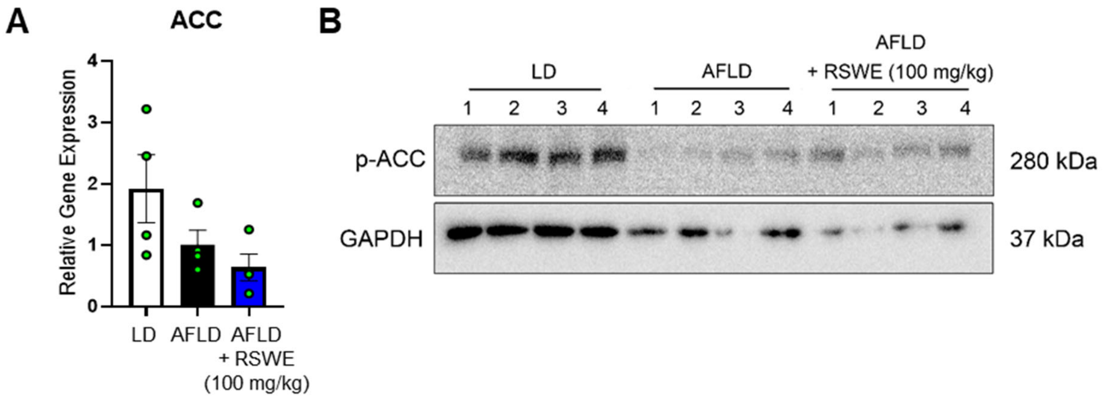

Figure S3. Effect of RSWE on ACC in liver of C57BL/6J NIAAA mouse model.

(A) The mRNA expression of ACC in the liver was analyzed using qPCR. (B) Protein levels of p-ACC was analyzed using Western blot analysis. GAPDH was used as a loading control. All data are expressed as the mean ± S.E.M. of data (*n* = 4, per group). RSWE. RSWE, Raphani Semen water extract; LD, liquid diet; AFLD, alcoholic fatty liver diseases.
